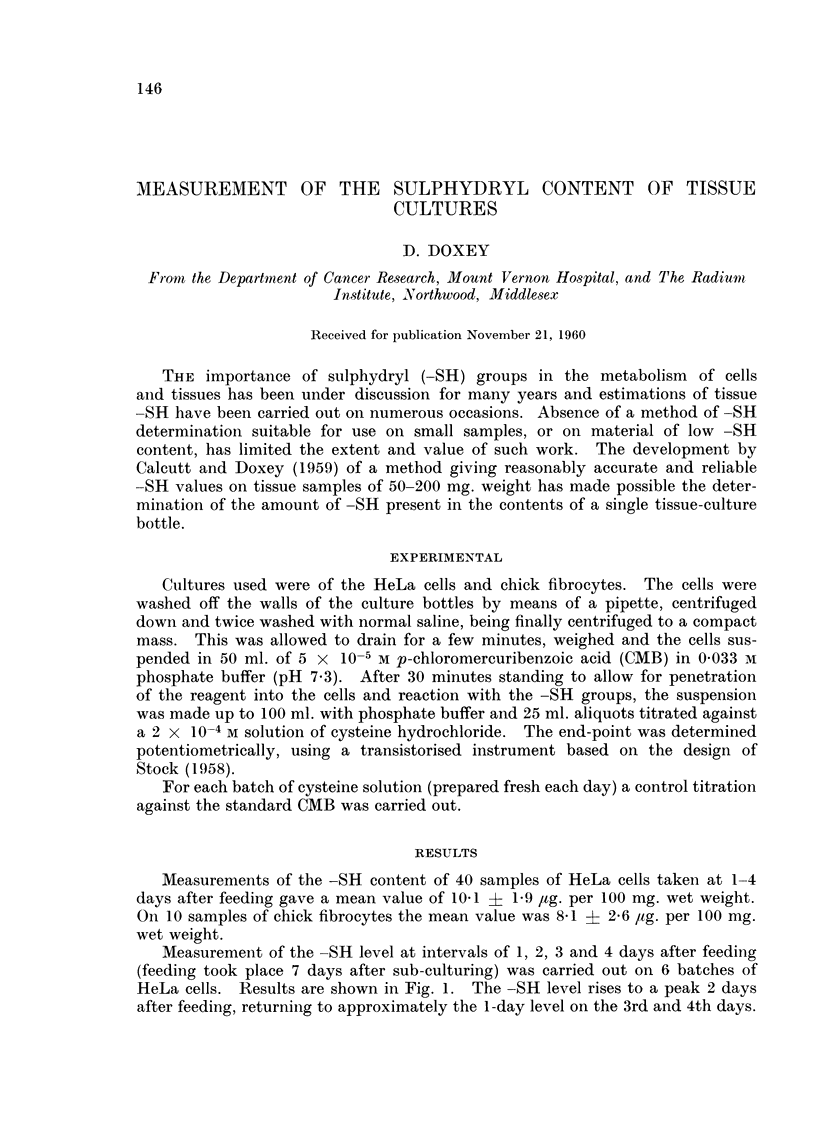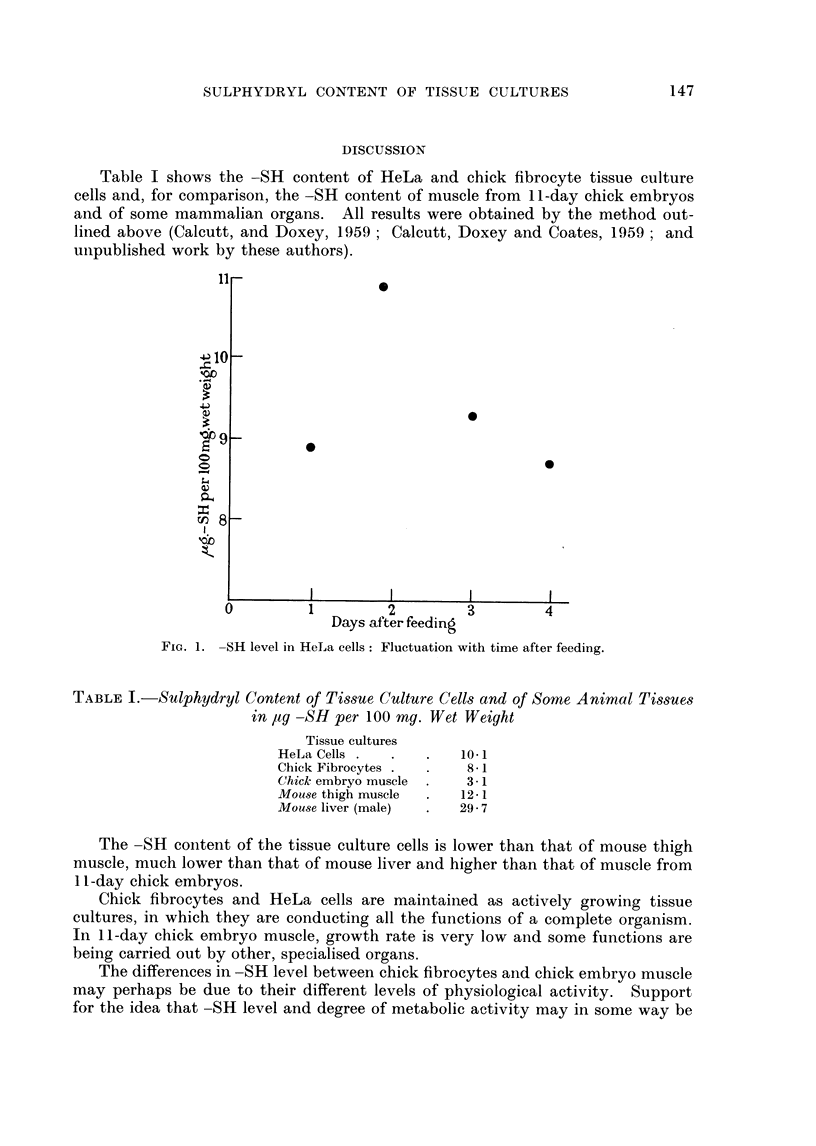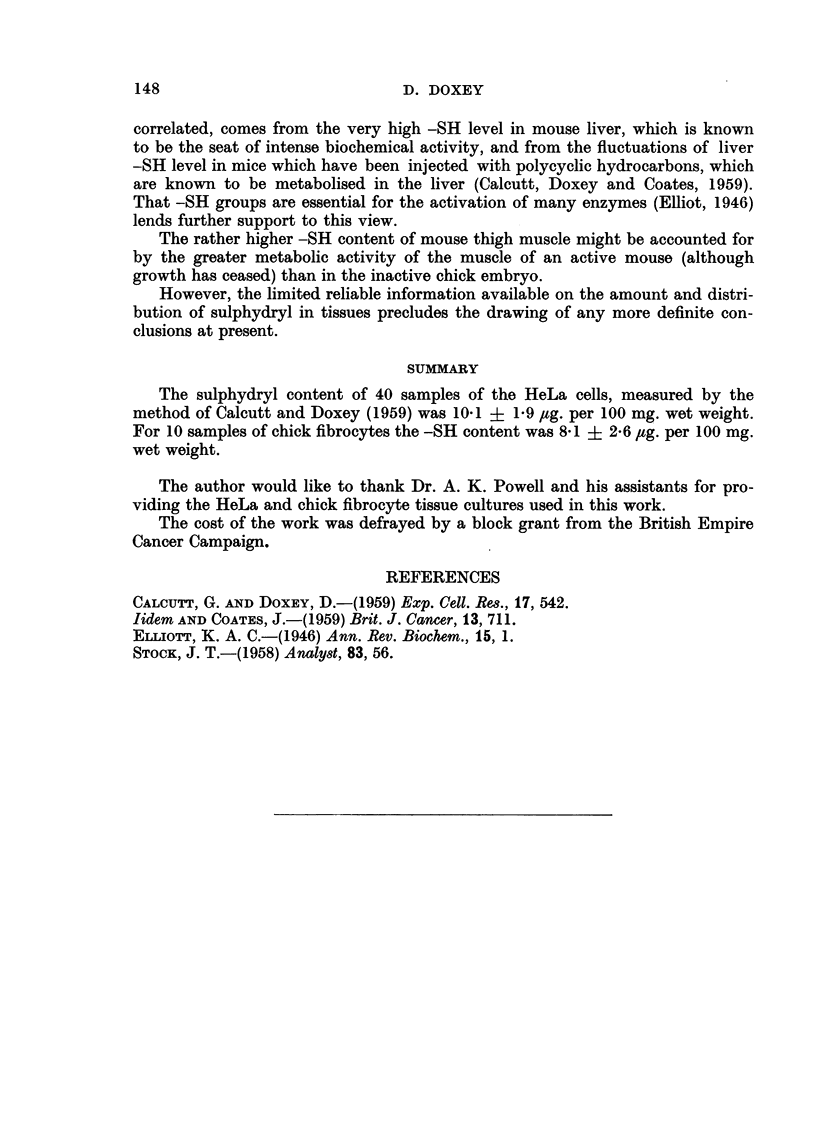# Measurement of the Sulphydryl Content of Tissue Cultures

**DOI:** 10.1038/bjc.1961.18

**Published:** 1961-03

**Authors:** D. Doxey


### MEASUREMENT OF THE SULPHYDRYL CONTENT OF TISSUE CULTURES

D. DOXEY

*From the Department of Cancer Research, Mount Vernon Hospital, and The Radium Institute, Northwood, Middlesex*

Received for publication November 21, 1960

THE importance of sulphydryl (–SH) groups in the metabolism of cells and tissues has been under discussion for many years and estimations of tissue –SH have been carried out on numerous occasions. Absence of a method of –SH determination suitable for use on small samples, or on material of low –SH content, has limited the extent and value of such work. The development by Calcutt and Doxey (1959) of a method giving reasonably accurate and reliable –SH values on tissue samples of 50–200 mg. weight has made possible the determination of the amount of –SH present in the contents of a single tissue-culture bottle.

### EXPERIMENTAL

Cultures used were of the HeLa cells and chick fibrocytes. The cells were washed off the walls of the culture bottles by means of a pipette, centrifuged down and twice washed with normal saline, being finally centrifuged to a compact mass. This was allowed to drain for a few minutes, weighed and the cells suspended in 50 ml. of $5 \times 10^{-5}$ M *p*-chloromercuribenzoic acid (CMB) in 0·033 M phosphate buffer (pH 7·3). After 30 minutes standing to allow for penetration of the reagent into the cells and reaction with the –SH groups, the suspension was made up to 100 ml. with phosphate buffer and 25 ml. aliquots titrated against a $2 \times 10^{-4}$ M solution of cysteine hydrochloride. The end-point was determined potentiometrically, using a transistorised instrument based on the design of Stock (1958).

For each batch of cysteine solution (prepared fresh each day) a control titration against the standard CMB was carried out.

### RESULTS

Measurements of the –SH content of 40 samples of HeLa cells taken at 1–4 days after feeding gave a mean value of $10·1 \pm 1·9$ $\mu$g. per 100 mg. wet weight. On 10 samples of chick fibrocytes the mean value was $8·1 \pm 2·6$ $\mu$g. per 100 mg. wet weight.

Measurement of the –SH level at intervals of 1, 2, 3 and 4 days after feeding (feeding took place 7 days after sub-culturing) was carried out on 6 batches of HeLa cells. Results are shown in Fig. 1. The –SH level rises to a peak 2 days after feeding, returning to approximately the 1-day level on the 3rd and 4th days.

### DISCUSSION

Table I shows the –SH content of HeLa and chick fibrocyte tissue culture cells and, for comparison, the –SH content of muscle from 11-day chick embryos and of some mammalian organs. All results were obtained by the method outlined above (Calcutt, and Doxey, 1959 ; Calcutt, Doxey and Coates, 1959 ; and unpublished work by these authors).

FIG. 1. –SH level in HeLa cells : Fluctuation with time after feeding.

TABLE I.—*Sulphydryl Content of Tissue Culture Cells and of Some Animal Tissues in μg –SH per* 100 *mg. Wet Weight*

| | |
|---|---|
| Tissue cultures | |
| HeLa Cells . . . | 10·1 |
| Chick Fibrocytes . . | 8·1 |
| *Chick* embryo muscle . | 3·1 |
| *Mouse* thigh muscle . | 12·1 |
| *Mouse* liver (male) . | 29·7 |

The –SH content of the tissue culture cells is lower than that of mouse thigh muscle, much lower than that of mouse liver and higher than that of muscle from 11-day chick embryos.

Chick fibrocytes and HeLa cells are maintained as actively growing tissue cultures, in which they are conducting all the functions of a complete organism. In 11-day chick embryo muscle, growth rate is very low and some functions are being carried out by other, specialised organs.

The differences in –SH level between chick fibrocytes and chick embryo muscle may perhaps be due to their different levels of physiological activity. Support for the idea that –SH level and degree of metabolic activity may in some way be correlated, comes from the very high –SH level in mouse liver, which is known to be the seat of intense biochemical activity, and from the fluctuations of liver –SH level in mice which have been injected with polycyclic hydrocarbons, which are known to be metabolised in the liver (Calcutt, Doxey and Coates, 1959). That –SH groups are essential for the activation of many enzymes (Elliot, 1946) lends further support to this view.

The rather higher –SH content of mouse thigh muscle might be accounted for by the greater metabolic activity of the muscle of an active mouse (although growth has ceased) than in the inactive chick embryo.

However, the limited reliable information available on the amount and distribution of sulphydryl in tissues precludes the drawing of any more definite conclusions at present.

### SUMMARY

The sulphydryl content of 40 samples of the HeLa cells, measured by the method of Calcutt and Doxey (1959) was 10·1 $\pm$ 1·9 $\mu$g. per 100 mg. wet weight. For 10 samples of chick fibrocytes the –SH content was 8·1 $\pm$ 2·6 $\mu$g. per 100 mg. wet weight.

The author would like to thank Dr. A. K. Powell and his assistants for providing the HeLa and chick fibrocyte tissue cultures used in this work.

The cost of the work was defrayed by a block grant from the British Empire Cancer Campaign.

### REFERENCES

Calcutt, G. and Doxey, D.—(1959) *Exp. Cell. Res.*, **17**, 542.
*Iidem* and Coates, J.—(1959) *Brit. J. Cancer*, **13**, 711.
Elliott, K. A. C.—(1946) *Ann. Rev. Biochem.*, **15**, 1.
Stock, J. T.—(1958) *Analyst*, **83**, 56.